# Research on Climate and Dengue in Malaysia: A Systematic Review

**DOI:** 10.1007/s40572-016-0078-z

**Published:** 2016-03-02

**Authors:** Yien Ling Hii, Rafdzah Ahmad Zaki, Nasrin Aghamohammadi, Joacim Rocklöv

**Affiliations:** Epidemiology and Global Health, Department of Public Health and Clinical Medicine, Umea University, Umea, Sweden; Julius Centre University of Malaya, Department of Social and Preventive Medicine, University of Malaya, Kuala Lumpur, Malaysia; Centre for Occupational and Environmental Health, Department of Social and Preventive Medicine, University of Malaya, Kuala Lumpur, Malaysia

**Keywords:** Dengue, DHF, Climate, Weather, Early warning, Malaysia

## Abstract

**Background & Objectives:**

Dengue is a climate-sensitive infectious disease. Climate-based dengue early warning may be a simple, low-cost, and effective tool for enhancing surveillance and control. Scientific studies on climate and dengue in local context form the basis for advancing the development of a climate-based early warning system. This study aims to review the current status of scientific studies in climate and dengue and the prospect or challenges of such research on a climate-based dengue early warning system in a dengue-endemic country, taking Malaysia as a case study.

**Method:**

We reviewed the relationship between climate and dengue derived from statistical modeling, laboratory tests, and field studies. We searched electronic databases including PubMed, Scopus, EBSCO (MEDLINE), Web of Science, and the World Health Organization publications, and assessed climate factors and their influence on dengue cases, mosquitoes, and virus and recent development in the field of climate and dengue.

**Results & Discussion:**

Few studies in Malaysia have emphasized the relationship between climate and dengue. Climatic factors such as temperature, rainfall, and humidity are associated with dengue; however, these relationships were not consistent. Climate change projections for Malaysia show a mounting risk for dengue in the future. Scientific studies on climate and dengue enhance dengue surveillance in the long run.

**Conclusion:**

It is essential for institutions in Malaysia to promote research on climate and vector-borne diseases to advance the development of climate-based early warning systems. Together, effective strategies that improve existing research capacity, maximize the use of limited resources, and promote local-international partnership are crucial for sustaining research on climate and health.

## Introduction

Dengue is one of the most rapidly spreading viral diseases in the world, despite increasing efforts to curb or reverse the upward trend. Moreover, geographical expansion of dengue from urban to suburban or rural settings has been observed in recent decades. The disease is caused by dengue viruses (DENV 1–4), which are transmitted to human hosts by *Aedes* mosquitoes. The dynamics of dengue transmission are influenced by multiple complex risk factors including host immunity, vector capacity, circulating DENV, weather or climate, dengue control capacity, and population movement. Climate variables influence dengue epidemiology through their indirect impacts on the biological aspects of mosquitoes and on incubation periods of DENV within mosquitoes (EIP) [1, 2, 3•, 4]. Climate change will alter the spatial and temporal dynamics of DENV ecology, potentially by increasing the vector’s flying range, increasing the duration of vector activity, and shortening the EIP [1]. Mechanistic dengue models have demonstrated that even small changes in EIP can have a large impact on dengue cases [5].

**Fig. 1:**
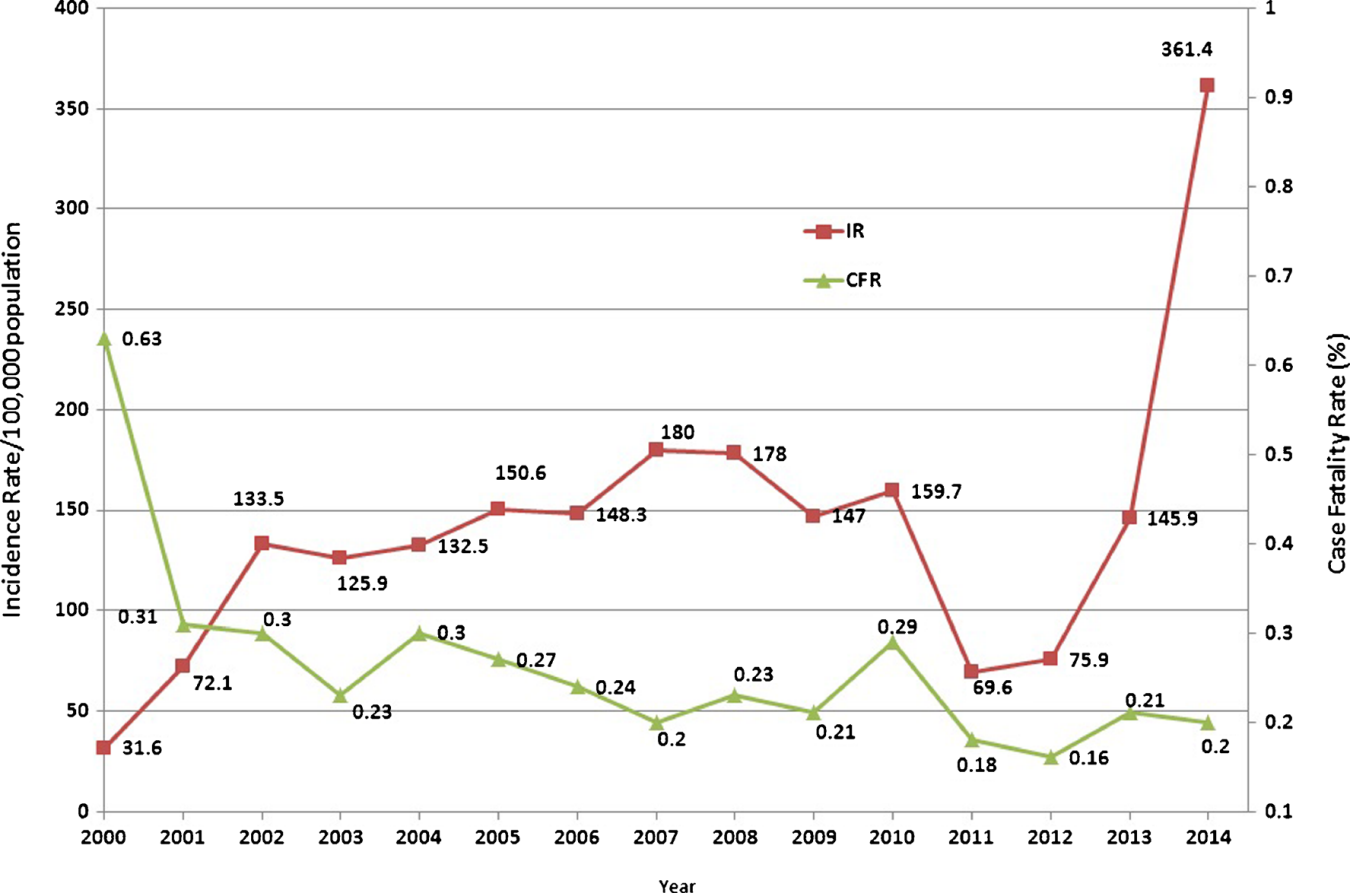
Dengue incidence rate and case fatality rate for Malaysia, 2000–2014 (source of data: Ministry of Health, Malaysia)

Dengue has been endemic in Malaysia since the 1970s, with increasing intensity and magnitude of outbreaks in recent decades [6]. The national incident rate (IR) increased from 32 cases per 100,000 population in 2000 to 361 cases in 2014, though a temporary reverse trend was observed in 2011 and 2012 (Fig. 1) [6]. At the same time, the case fatality rate decreased from 0.6 % in 2000 to 0.2 % in 2014. In early 2015, dengue cases reached or exceeded the number of cases reported during the epidemic period in 2014 [6]. However, this may have been partly due to a spillover from the outbreak in 2014. All four serotypes of dengue viruses coexist in Malaysia, with each of DENV1–3 being the predominant virus in different periods, while DENV4 exerts less influence in the country [6]. A recent study suggested that the dengue outbreaks in Malaysia during 2013 and 2014 were caused by changes in DENV serotypes 4 to 6 months prior to each outbreak [6].

In 2011, the Ministry of Health implemented a National Dengue Strategic Plan (NDSP) to increase dengue control efforts. The NDSP employed strategies to enhance dengue surveillance, vector control, case and outbreak management, population mobilization, and research in innovative dengue control tools and strategies [6]. Subsequently, a short-lived downward trend of dengue IR was observed in 2011 and 2012. However, the country has experienced a surge in dengue IR since 2013. A study by Ng et al. in 2015 suggested that the 2013 outbreak in Malaysia was probably caused by a switch in predominant dengue serotype from DENV3 and DENV4 to DENV2 [7].

Effective vector or mosquito control measures are critical to achieving and sustaining a reduction in morbidity attributable to dengue [8]. Vector control is aimed at disrupting dengue transmission in order to reduce the incidence of infection and consequently prevent outbreaks. Vector control, however, can be capital- and labor-intensive. According to a study in Singapore, the cost for dengue control could be around 42–59 % of the total economic burden of dengue [9]. An effective early warning system will enhance outbreak preparedness and response, an important element in planning of early intervention and resource allocation [8]. Thus, an early warning of dengue outbreak not only enhances dengue control but also reduces the health and economic burden of dengue in the population. In the past decade, studies have shown evidence of a relationship between climate and dengue and of the feasibility of using climate data to predict dengue outbreaks [10–15]. These studies contribute to the advanced development of climate-based dengue forecast modeling that could pave the way for an early warning system. Climate data is freely and publicly available. Given the well-documented relationship between climate and dengue, climate could be an economical, simple, and effective tool for predicting outbreaks, especially in developing or resource-constrained countries.

Dengue cases are reported primarily in developing countries with restricted healthcare resources. This study aims to review scientific studies on climate and dengue and the prospect and challenges of future research in climate-based dengue early warning systems in Malaysia, a developing and dengue-endemic country situated in the dengue epidemic center of Southeast Asia.

## Methodology

We systematically reviewed evidence reported from 1990 to 2015 for an association between climate and dengue in Malaysia based on scientific findings derived from statistical modeling of cases as well as laboratory and field studies on *Aedes* mosquitoes and DENV. In September and October 2015, we reviewed the literature in the following electronic databases: 1) PubMed, 2) Scopus, 3) EBSCO (MEDLINE), 4) Web of Science, and 5) the World Health Organization publication databases, namely WHOLIS and WHO IMSEAR. We searched each of the databases using a combination of keywords including “dengue,” “climate,” “weather,” “temperature,” “rainfall,” “humidity,” and “Malaysia.” The inclusion criteria included peer-reviewed original papers reporting studies on the links between climate variables and dengue in Malaysia, published in international or national journals from 1990 to 2015, in English, and retrieved from one of the above-mentioned electronic databases. We identified 127 publications (including duplicate articles) using data in Malaysia. In the first review, titles and abstracts were screened based on inclusion criteria to select articles that analyzed the relationship between climate and dengue. Laboratory and field studies on climate and *Aedes aegypti*, *Aedes albopictus*, and DENV were also included. Full articles were reviewed in the second round of selection. After full-text review and removal of duplicate articles, nine original papers were selected for this review.

**Fig. 2:**
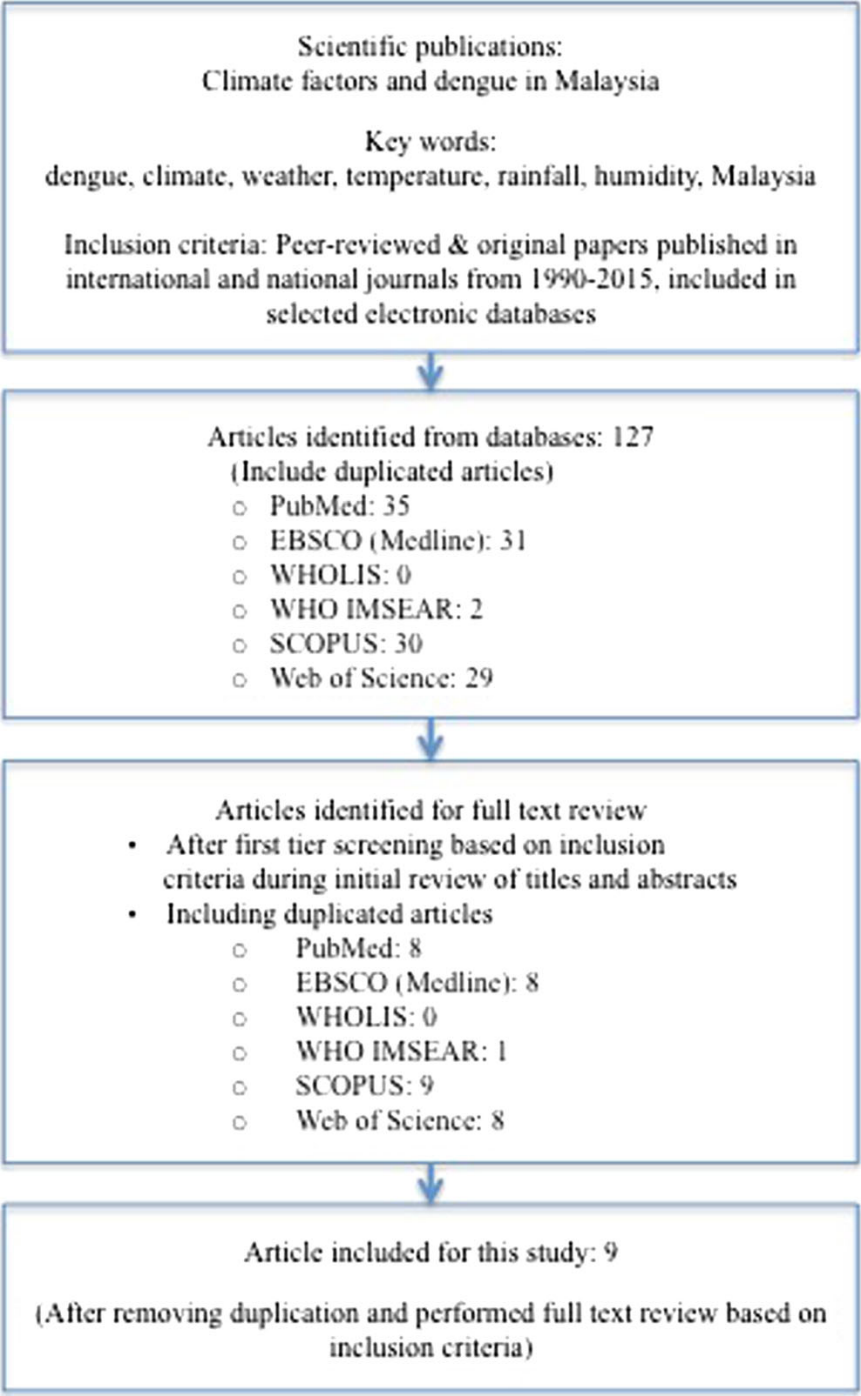
Summary of strategies, process, and results of literature search

A summary of the search strategy and results is presented in a flowchart (Fig. 2). The characteristics of selected articles, including choice of climate factors for each study, are shown in Table 1. Methodologies and key findings of the studies are presented in Table 2 according to publication chronology and types of dependent variables. Dependent variables are arranged in three categories and coded with letters representing types of studies: C denotes dengue cases, V indicates vector or *Aedes* mosquitoes, and D represents the dengue virus. Findings and discussion focused on the establishment of links between climate and dengue and the progress of research in climate-based dengue early warning.

**Table 1:** Characteristics of selected publications

Publication			Study area		Data		Dengue			Climate variables							Non-climatic variables		
Article ID	Title & Author(s)	Year	State	Study Site	Time Frame	Resolution	Cases	Vector	DENV	Mean temp	Max temp	Min temp	Rainfall	Humidity	Cloud	Wind	Past cases	Lag	Trend/Season
C1	Testing the impact of virus importation rates and future climate change on dengue activity in Malaysia using a mechanistic entomology and disease model C.R. Williams et al. [23]	2015	Selangor	Petaling	2007–2012	Day	■	■		■								■	
C2	Assessing weather effects on dengue disease in Malaysia Cheong YL et al. [24]	2013	Federal Capital Selangor	Kuala Lumpur, Putrajaya	2008–2010	Day	■			■	■	■	■	■		■		■	■
C3	Coupling of remote sensing data and environment-related parameters for dengue transmission risk assessment in Subang Jaya, Malaysia N. Che Dom et al. [25]	2012	Selangor	Subang Jaya	2006–2010	Day Week Month	■			■	■		■	■					
C4	Modelling dengue fever (DF) and dengue haemorrhagic fever (DHF) outbreak using Poisson and negative binomial model Wan Fairos et al. [16]	2010	Federal Capital, Selangor	Hospital Putrajaya	July 2006–Dec 2008	Day	■				■	■	■	■	■	■		■	
V1	Relationship between rainfall and *Aedes* larval population at two insular sites in Pulau Ketam, Selangor, Malaysia Wee L.K. et al. [26]	2013	Selangor	2 townships in Pulau Ketam	Oct 2007–Oct 2008	Day		■		■			■						
V2	*Aedes* larval population dynamics and risk for dengue epidemic in Malaysia Rohani et al. [17]	2011	Kuala Lumpur, Pahang, Kedah, Johor	Residential estates	Oct 2007–June 2009	Week	■	■		■			■	■			■		
V3	Life tables study of immature *Aedes albopictus* (Skuse) (Diptera: Culicidae) during the wet dry seasons in Penang, Malaysia Hashim et al. [27]	2008	Penang	Lurah Burung, University Sains Malaysia	Jan–Dec 2003	Day		■		■			■	■					
V4	Seasonal abundance of *Aedes albopictus* in selected urban and suburban areas in Penang, Malaysia Rozilawati et al. [28]	2007	Penang	Residential estates	Mar 2003 –Mar 2004	Week		■		■			■	■				■	
D1	The effect of extrinsic incubation temperature on development of dengue serotype 2 and 4 viruses in *Aedes aegypti* (L.) Rohani A et al. [29]	2009	Kuala Lumpur, Selangor	Medical Entomology Unit, Institute for Medical Research	–	Day			■	■									

(Legends: C = Case, V = Vector, D = DENV)

**Table 2:** Methods and key findings of selected publications

Article ID	Analysis methods	Main findings
C1	Dengue models CIMSiM and DENSiM were used to simulate the impacts of: - Virus importation on dengue incidence - Temperature increase on dengue incidence	A moderate increase in temperature did not necessarily lead to an increase in dengue cases. Virus importation rate was correlated to dengue incidence.
C2	- Correlation analysis between climate variables and dengue cases - Poisson generalized additive model with natural cubic splines was used to assess relationship between weather parameters and dengue cases. Stepwise forward and backward for variable selection, lag terms and seasonal trend were considered, AIC for model selection, and sensitivity was performed with degrees of freedom ranging from 3 to 7. Distribution lag non-linear models were used to assess the delayed effects of weather parameters on dengue cases.	Min temperature and dengue exhibited a non-linear positive relationship, with highest risk of dengue occurred at 25.4 to 26.5 °C at a lag of 51 days. Rainfall increased risk of dengue at a lag of 30 days with sharp increase of risk when bi-weekly cumulative rainfall exceeded 200 mm. Wind speed increased risk of dengue on current day and with wind speed at 3–5 knots at lag of 1–3 months, inverse effects on the risk of dengue occurred with wind speed at up to 3 knots and at 5 knots and above.
C3	- Correlation analysis to assess relationship between climate factors and dengue incidence - Remote sensing application software for analysis of land-use and dengue incidence	Temperature was correlated with dengue incidence. Rainfall was an important factor influencing the trend of dengue outbreak. Humidity was not a contributing factor of dengue outbreak. Land-use pattern and housing types were related to the distribution of dengue incidence.
C4	- Poisson and negative binomial regression models were developed with lag periods ranging from 7 to 28 days. Significance tests were performed using max likelihood ratio statistics (Deviance statistics) to determine the goodness of fitted models.	Minimum temperature increased the risk of dengue cases by 21.09 % at lag of 14 days and 14.25 % at lag of 21 days. Humidity reduced the risk of dengue cases by 1.57 % at lag of 14 days. Wind speed reduced the risk of dengue cases by 18.71 % at lag of 14 days and about 17 % at lag of 21 days.
V1	- Ovitrap surveillance was conducted according to guidelines of the Ministry of Health, Malaysia; 80 ovitraps placed indoors and outdoors at randomly selected locations. GPS was used to monitor ovitrap locations. *Aedes* larvae were counted and identified at the third or fourth instar using a compound microscope. - Independent *t*-test was used to evaluate correlation between larval distribution and trap sites.Correlation analyses were performed to assess relationship between rainfall and ovitrap indices.	Study area 1: Kampung Pulau Ketam Correlation between rainfall and *Ae. aegypti* indoor ovitrap indices and outdoor ovitrap indices peaked at lag 35 days (r=0.54) and lag 25 (r=0.45) days, respectively. Peak correlation between rainfall and outdoor *Ae. albopictus* ovitrap index occurred at lag 25 days (r=0.72). Study area 2: Kampung Sungai Lima Correlation between rainfall and indoor *Ae. aegypti* ovitrap indices peaked at lag 27 days (r=0.54), and correlation between rainfall and outdoor *Ae. aegypti* ovitrap inidices peaked at lag 28 days (r=0.63) and 44 days (r=0.66).
V2	- Ovitraps surveillance outside occupied houses at residential estates three states and the capital city. Mosquito larvae were identified using standard taxonomic keys. - Pearson correlation and autoregressive distributed lag (ADL) model were used to analyze the relationship between climate factors and vectors and dengue.	Larvae: Rainfall was correlated with larval population in all study areas, with correlation coefficients ranging from −0.19 to 0.24 at lag week 0 and from 0.42 to 0.57 at lag week 1. Min and max temperature influenced larvae, with correlation coefficients ranging from −0.41 to −0.81 and 0.23 to 0.54, respectively. Max humidity was correlated with larval density, with coefficients ranging from 0.32 to 0.85. Dengue cases: Rainfall influenced the risk of dengue cases across all study areas (Kedah, Pahang, Johor, and Kuala Lumpur) at lag of 1 week. Min temp exhibited inverse relationship with dengue cases in Kedah, Pahang & Johor at lag week 0, while it posed positive effects on the risk of dengue in Kedah and negative impact on the risk of dengue in Pahang at lag of 1 week. Max temp increased risk of dengue cases in Kedah & Pahang, and reduced risk of cases in Kedah at lag of 1 week. Maximum humidity increased risk of dengue cases in all study areas.
V3	- Ovitrap surveillance was conducted to observe *Aedes albopictus* development time and mortality at aqua stage. - Kruskal–Wallis and correlation analysis using SPSS	Mean temperature was correlated with larval development time (*r* = 0.64) but not mortality. Rising temperature reduced larval development time. Immature *Aedes albopictus* completed development (aqua stage) in an average 6.82 and 8.14 days in dry and wet season, respectively. Rainfall showed no effect on larval development time and mortality.
V4	- Ovitrap surveillance was conducted in an urban and a suburban area. - Pearson correlation analysis was preformed to evaluate relationship between ovitrap index, mean number of eggs and weather parameters.	Study area 1: Taman Permai Indah Mean number of mosquito eggs was strongly correlated with rainfall at lag week 1 (*r* = 0.98) and 2 (*r* = 0.77) and with humidity (r = 0.48) it was negatively correlated with mean temperature (*r* = -0.37). Study area 2: Kampung Pasir Gebu, Penanga Rainfall and mean number of eggs (*r* = 0.92) were positively correlated, whereas mean temperature and humidity showed no effect. Rainfall was correlated with ovitrap (*r* = 0.39)
D1	- Laboratory experiments. Engorged mosquitoes were retained at selected temperature to observe dengue virus development in *Aedes* vectors. Virus detection was carried out by RT-PCR.	DENV-2 and DENV-4 increased replication rates and shortened incubation at higher temperatures. Mosquitoes infected with DENV-2 or DENV-4 at day 5 when temperature was at 30 °C and infected at day 9 when temperature was at 26 °C or 28 °C. At 26 °C or 28 °C, max length of incubation period was 21 days for DENV-2 and 23 days for DENV-4; at 30 °C, max incubation period was 19 days for both viruses.

Legend: C = case, V = vector, D = DENV

## Results

Almost all the studies used data from two of the 13 states in Peninsular Malaysia, namely Selangor and Penang, and the majority of papers were published after 2010 (Table 1). Temperature, rainfall, and humidity were the key climate variables used in almost all the papers, whereas two included wind speed and one considered clouds. Statistical analyses were performed using daily or weekly data over study periods ranging from 1 to 6 years. Four papers evaluated the relationship between climate variables, measured as °C and mm, and dengue cases based on regression models, while others performed correlation analyses of climate variables, measured as °C and mm, and larvae population density or incubation rate of DENV. Only two papers used advanced statistical modeling for analyses of non-linear relationships between climate and dengue.

As shown in Table 2, article C1 found no significant relationship between increasing temperature and dengue cases, while article C3 suggested that temperature and rainfall significantly influenced dengue trends. Articles C2, C4, and V2 reported a positive relationship between minimum temperature and dengue cases at lag periods ranging from 7 to 51 days. Wind speed at different ranges of knots posed different risk levels. Findings of the effects of humidity and rainfall on dengue cases were inconsistent. Cheong et al. (article C2) reported that the highest risk of dengue cases occurred within a small temperature range, from 25.4 to 26.5 °C, at a lag of 51 days. Bi-weekly cumulative rainfall and dengue exhibited a linear relationship at a lag of 30 days. Similarly, Wan Fairos et al. (article C4) reported that minimum temperature (21–26 °C) was associated with an increase in dengue cases at a shorter lag period of 14 and 21 days, and an increase in humidity reduced the number of cases at a lag of 14 days [16]. Rohani et al. (article V2) also documented that rainfall, temperature, and humidity were associated with dengue cases at a lag of up to 1 week; however, the findings showed dissimilar effects of climate on dengue incidence among different study areas [17].

Articles V1 and V2 showed that rainfall was positively associated with larval population at a lag of 7 to 44 days, but article V3 reported that rainfall posed no effect on larval development time or mortality rate. Article V2 suggested maximum temperature and maximum humidity increased larval populations, while minimum temperature posed negative effects on larval population. Article V3 reported rising mean temperature reduced larval development time. Article V4 showed that the mean number of *Aedes* eggs increased 1–2 weeks after rainfall. Humidity was positively associated with the mean number of eggs, while mean temperature suggested reverse effects. Article V4 also reported different results for the effects of mean temperature and humidity on mean number of eggs between two study areas. Laboratory experiments suggested that a rise in temperature from 26 to 30 °C increased the replication rate of DENV–2 and DENV–4 and reduced the EIP in mosquitoes from 9 to 5 days (article D1). The maximum length of the EIP was prolonged from 19 to 23 days for DENV–2 and DENV–4 at 26 °C.

## Discussion

Malaysia is a tropical country with warm temperatures, high humidity, and copious rainfall. The average temperature is around 27 °C, and mean cumulative rainfall is about 2500 mm a year. The warm and wet weather coupled with high humidity is conducive to the development of *Aedes* mosquitoes, viral replication, and transmission of dengue year-round [3•, 18••, 19].

Our literature review revealed only a few studies in Malaysia that focused on climate and dengue. Moreover, studies on the development of climate-based early warning systems in a local context were not identified in our literature review. Climate-based dengue forecast modeling thus far has generally been geographically bound, though the framework of the model could be generalized to include multiple regions. This could be due in part to varying local climate conditions and the influence of multiple non-climatic factors unique to the respective study area. Thus, scientific studies on climate and dengue using local data are necessary for analyses of the relationship and for developing a climate-based dengue early warning system. Numerous studies in recent decades have reported links between climate, dengue, *Aedes* mosquitoes, and DENV. A systematic review in Brazil (2013) suggested that about 580 publications worldwide had reported findings in climate and dengue, and that 31, or 5.3 %, of these publications were from Brazil [20]. Our literature search suggested that Malaysia contributed about 1.4 % of total publications as of 2013.

A majority of the studies in Malaysia indicated an association between dengue, rainfall, and temperature, with lag periods ranging from 7 to 51 days. This lag term usually serves as a window or lead-time for vector control. Optimal lead-time for early warning could vary according to factors such as the capacity of vector control and the effectiveness of outbreak management in the respective study areas. A study in Singapore has suggested that a 3-month window would provide sufficient time for effective mitigation [21]. Aside from climate variables, the dynamics of dengue transmission in a given study area are influenced by multiple complex factors including circulating dengue serotypes, herd immunity, population density, land-use, dengue control policy and capacity, outbreak control management, and community commitment to the removal of larvae in residential areas.

According to the Malaysia Meteorological Department, long-term mean temperature (comparing climate data for 1961–1990 and 1998–2007 obtained from various weather stations) increased by 0.5–1.5 °C and 0.5–1.0 °C in Peninsular Malaysia and East Malaysia, respectively [22]. A large temporal and spatial variation in rainfall was observed, and rainfall in East Malaysia was higher than that in Peninsular Malaysia. Based on a study of climate change scenarios for Malaysia from 2001 to 2099, the projected mean temperature would most likely increase by 1.0–3.5 °C and 1.1–3.6 °C for East and Peninsular Malaysia, respectively [22]. The study further reported that the projected trend in annual temperature anomaly would most likely rise linearly, and the average temperature anomaly over the period 2020–2029 was expected to increase by about 1.0 to 1.5 °C, depending on spatial variation. The cumulative rainfall projection varied according to year and location. At the same time, the estimated average annual rainfall anomaly for the period 2020–2029 was likely to decrease by 8.8 to 18.7 %, with changes varying according to locality [22]. Furthermore, regional temperature anomaly, El Niño and La Niña, exerts influence on local weather. The country experienced a severe dry spell during El Nino episodes in 1963, 1997, and 2002 [22]. In recent years, several episodes of massive, severe flooding events have occurred in multiple cities across the country as a result of extremely heavy rainfall in a short amount of time.

Considering the relationship between climate and dengue, climate projections imply an increasing risk of dengue outbreaks in Malaysia. In addition to strengthening surveillance and control systems with enhanced capacity, an epidemic prediction capability is needed to allow timely mitigation and effective resource deployment. To date, no climate-based early warning or outbreak alert system has been established for dengue surveillance in Malaysia. Lack of data and knowhow, as well as inadequate support from policy-makers for the use of such a system, could hamper the development of technological innovations to control dengue. Local to national dengue data is not publicly available in Malaysia. Researchers may be subjected to lengthy application processes in order to obtain data from respective health departments. Moreover, the reliability and validity of available data is also a challenge. Nevertheless, the quality of data regarding dengue has progressively improved since 2009.

The health authority can encourage studies on climate-related diseases by making dengue case data publicly available for research purposes and by supporting the development and integration of a climate-based dengue forecasting system through synchronized efforts across national dengue surveillance systems and meteorological departments. Support from academic and health institutions in providing advanced knowledge and technical skills for disease forecast modeling as well as funding resources will boost research capacity and interest. In addition, the inclusion of advanced disease modeling as a university course module for medical or allied health students and researchers would help to sustain research efforts in climate and dengue.

## Conclusion

In view of projected climate change and the effects of climate on dengue, the risk of dengue outbreak is likely to increase if effective dengue or vector control is not in place. It is imperative that institutions in Malaysia actively promote research and surveillance efforts around climate and dengue with sufficient coverage and depth to advance the development of and capitalize on a climate-based early warning to control the risk of dengue. Research topics including optimal lead-time for early warning, forecast modeling systems, sustainability of forecast precision and integration of climate into local surveillance systems are encouraged. Research capacity, data quality and availability, resource constraints, and insufficient support from public and private institutions all contribute to the current shortfall. Therefore, effective strategies to improve existing research capacity, maximize the use of limited resources, and promote public–private collaboration at the local, national, and international levels are crucial for the sustainability of local research in climate and dengue.
